# Current status of human endogenous retrovirus annotation

**DOI:** 10.1093/bib/bbag062

**Published:** 2026-02-16

**Authors:** Sergei Sinitsyn, Marharyta Klianitskaya, Michelle Vincendeau, Jan Pačes, Dmitrij Frishman

**Affiliations:** Department of Bioinformatics, TUM School of Life Sciences, Technical University of Munich, 85354 Freising, Germany; Institute of Molecular Genetics, Academy of Sciences of the Czech Republic, Vídeňská 1083, 14220 Prague, Czech Republic; Institute of Virology, School of Medicine, Technical University of Munich, 80333 Munich, Germany; Institute of Virology, Helmholtz Zentrum München, 81675 Munich, Germany; Institute of Molecular Genetics, Academy of Sciences of the Czech Republic, Vídeňská 1083, 14220 Prague, Czech Republic; Department of Bioinformatics, TUM School of Life Sciences, Technical University of Munich, 85354 Freising, Germany

**Keywords:** genome annotation, bioinformatics databases, genomic repeats, transposable elements, comparative genomics

## Abstract

Human endogenous retroviruses (HERVs) constitute a significant fraction of the human genome and are increasingly recognized for their roles in both physiological and pathological processes. Despite their biological importance, the annotation of HERV elements remains inconsistent across major public databases. In this study, we present a comprehensive comparative analysis of three key HERV annotation resources: DFAM, Human Endogenous Retroviruses Database (HERVd), and RepBase. We systematically examine their content, classification schemes, and postprocessing workflows and assess the concordance of their annotations based on genomic coordinates. Our analysis reveals substantial discrepancies in element counts, genome coverage, and repeat fragmentation strategies, which we trace back to differences in curation methodologies—ranging from DFAM’s hidden Markov model-based automated detection to HERVd’s semimanual defragmentation. Using refined matching criteria, we demonstrate that up to 93% of HERV records can be reconciled across databases, yet each source still contributes a substantial proportion of unique elements. We highlight the complementary strengths of these resources and provide practical recommendations for their usage in HERV research. Our findings underscore the need for harmonized standards in retroelement annotation and may inform future efforts toward unified and comprehensive HERV cataloging, particularly in light of emerging genome assemblies such as T2T-CHM13.

## Introduction

Human endogenous retroviral (HERV) elements are DNA sequences in the human genome that originate from ancient infections by exogenous retroviruses. The RNA of these retroviruses is reverse-transcribed into DNA, which then integrates into germline cells, allowing it to be passed down through Mendelian inheritance [1, 2]. During this transmission process, the viral genes accumulate mutations or undergo recombination [1, 3]. These mutated viral remnants have become a significant part of the human genome, making up ~8% of its total sequence [2–4] (estimates range from 5% to 14% across vertebrates) [5]. Historically, HERVs were often regarded as “junk DNA” or fossilized elements; however, there is now substantial evidence indicating that they have crucial roles in normal physiological processes as well as various pathological conditions [6]. As a result, interest in the research of HERVs is increasing, particularly regarding their implications in numerous human diseases, including cancer, infectious diseases, age-related conditions, inflammatory and autoimmune disorders, and neurological diseases. While their genomic characterization presents numerous technical challenges, advancements in technologies such as next-generation sequencing (NGS) have shown potential for detecting HERV insertions and polymorphisms in humans, leading to the development of computational tools for their analysis.

Classifying HERVs can be challenging due to significant changes over evolutionary time caused by mutations, recombinations, and deletions of internal sequences, often leaving only long terminal repeats (LTRs). Older repeats are generally harder to classify due to fragmentation and uncertainty at the time of discovery. To support the study and annotation of endogenous retroviruses, several curated databases have been developed, each offering valuable and complementary perspectives. The Human Endogenous Retroviruses Database (HERVd) [7] provides a manually curated collection of HERV sequences with detailed genomic context and classification, making it particularly useful for researchers seeking locus-specific information. Its strength lies in its curated entries and biological annotations that can be leveraged for targeted investigations. RepBase [8], a long-standing reference database for repetitive DNA, offers a comprehensive catalog of consensus sequences for a wide range of transposable elements, including HERV families. Its utility is especially evident in comparative genomics and repeat masking applications, where it serves as a foundational reference for identifying repetitive elements across multiple genomes. The most recent public version is currently available through the University of California, Santa Cruz (UCSC) Genome Browser [9], while ongoing updates are accessible via subscription. DFAM [10] is an open-access database of repetitive DNA families, sequence models, and genome annotations. It introduces a profile-based approach using hidden Markov models (HMMs), which enables sensitive detection of diverse and structurally variable repeat elements. This makes it well-suited for broad genome annotation and integration with tools such as RepeatMasker [11]. It also provides alignments and curated family models that facilitate both computational and functional studies.

Similarity to animal retroviruses (Classes I, II, and III) is the primary method for classifying HERVs. It is based on their sequence similarities to exogenous animal retroviruses. Class I families are similar in sequence to mammalian Gammaretroviruses (type C) and Epsilonretroviruses (Type E). Class II families show homology to mammalian Betaretroviruses (Type B) and Deltaretroviruses (Type D). Class III families are similar to foamy viruses. These three classes correspond to the three major branches found in reverse transcriptase-based phylogenies of retroviruses and endogenous retroviruses (ERVs).

Within these classes, HERVs are further grouped into superfamilies if homologies are well conserved across the gag, pro, and env viral genes. These superfamilies can be further subdivided into groups. The HERVd, for example, assigns elements to one of five top-level superfamilies: ERV1, ERV2, ERV3, Gypsy, and Unclassified, which is consistent with the classification described in Kojima’s article [12]. ERV1 corresponds to Class I, ERV2 to Class II, and ERV3 to Class III.

Historically, human ERVs were often designated with “HERV” followed by a capital letter, such as K, L, or W. While attempts at standardized nomenclature exist, naming has historically been less uniform. Families can be named based on the exogenous retrovirus they resemble, the priming tRNA used during reverse transcription (like HERV-W, HERV-K), a neighboring gene (HERV-ADP), a clone number (HERV-S71), or an amino acid motif (HERV-FRD). RepBase uses a format involving the superfamily, subgroup, a number, and species abbreviation, though not all entries follow this rule.

Despite the availability of established HERV databases, there is currently no universally accepted framework for classifying or annotating HERVs. Terminology, grouping, and annotation strategies vary considerably between databases, often resulting in discrepancies in nomenclature and coverage. The lack of standardization hampers comparative analysis and limits the ability to draw reliable biological conclusions. The aim of this study was to systematically compare the three main resources on which most HERV analyses are currently based: RepBase, DFAM, and HERVd. We examine each database’s content, curation workflow, and postprocessing pipeline and trace how these decisions shape the annotation of the major HERV superfamilies (ERV1, ERV2, ERV3, and Gypsy-like elements). By providing this comparative roadmap, we aim to help researchers select the most appropriate set of benchmarks for their specific purposes and accelerate ongoing efforts to achieve harmonized standards in HERV research.

## Materials and methods

### Data sources

Information about HERV elements was obtained from three databases: HERVd [7], DFAM [10], and RepBase [8] as available from the RepeatMasker [11] track of the UCSC Genome Browser [9]. We benchmarked only resources that (i) provide genome-coordinate, locus-resolved HERV annotations for human reference, (ii) are publicly accessible with stable releases/snapshots and bulk download, and (iii) permit reuse for research. Under these criteria we included HERVd and RepeatMasker-derived coordinates built from Dfam/RepBase libraries. Ensembl repeat annotation, being a redistribution of RepeatMasker outputs, was not treated as an independent source. The detailed workflow of data collection is presented in Supplementary File 1. Using RepeatMasker based on DFAM 3.9, the total of 5 670  831 human repeat elements were downloaded and 782 639 DFAM entries of the type LTR were retained. This annotation was kindly provided by Mr Robert Hubley and will soon be available on the RepeatMasker page.

We also downloaded 720 177 LTR repeats from RepBase via the UCSC SQL-server. Note that the RepBase data available via the UCSC Genome Browser corresponds to the last publicly available release (“RELEASE 20130422”) dating back to 2013 and is thus not up to date. The RepBase annotation of ERVs is inconsistent in that in most cases only the superfamily assignment is provided, while in some other cases, they are described by family names. Furthermore, for 3438 LTR elements, no further classification is given. For these reasons, we employed the information from the DFAM database to classify LTR elements from RepBase, using the mapping between the two databases available via the DFAM API (keyword “RepBase_name”). Both DFAM and RepBase annotation is generated by RepeatMasker, but it is important to note that different mapping and alignment strategies were used in the calculations: “RMBlast” [13] for RepBase and “nhmmer” [14] for DFAM.

HERVd (version 3) provides information on ERV repeats in a separate file such that no additional postprocessing or improvement was required; this file also contains qualitative characteristics of these records, in particular the “completeness” parameter, and this information is unique in this database. The degree of degradation is defined as the percentage of nucleotides lost in the entry compared to the consensus sequence from the DFAM database. The final datasets of HERV elements used in this study contained 565 471, 782 639, and 720 177 records obtained from the HERVd, DFAM, and RepBase databases, respectively (see Data Availability).

### Annotation of human endogenous retroviral elements

From each database, HERV annotation was obtained, including the ID and coordinates of each HERV locus, as well as of internal portions and LTRs of HERV elements. HERV elements were classified into five superfamilies (ERV1, ERV2, ERV3, Gypsy, Unclassified) as described in our previous study [15]. Briefly, all data sources do not provide any information on the association between the internal portions of the proviruses, their LTRs, and superfamilies. To establish such an association, we used the data from this [12] paper as a basis, in which the five superfamilies are further subdivided into 22 groups. For example, the ERV3 superfamily contains the following groups: HERVL, HERVS, MaLR, and Unclassified. Within each group, a list of internal portions and their associated LTRs is provided, some of the remaining ones were annotated using DFAM, and two types of internal regions not associated with any LTRs and 122 LTR types not associated with any internal regions were ignored.

### Comparison between three sources of annotation

To find matching elements between the HERVd, DFAM, and RepBase databases, we compared their chromosomal locations and identified elements overlapping by at least 1 bp using the PyRanges package [16]. Subsequently for each pair of overlapping elements s_1_ and s_2_ segment overlap value *Sov* [17] was calculated as follows:


$$ Sov=\frac{minov\left({s}_1,{s}_2\right)+\delta \left({s}_1,{s}_2\right)}{maxov\left({s}_1,{s}_2\right)}, $$

where *minov (s_1_, s_2_)* is the overlap between *s*_1_ and *s*_2_, *maxov (s_1_,s_2_)* is the total extent of *s*_1_ and *s*_2_, and δ is defined as follows:


$$ \delta =\textrm{min}\left\{\begin{array}{c} maxov\left({s}_1,{s}_2\right)- minov\left({s}_1,{s}_2\right)\\{} minov\left({s}_1,{s}_2\right)\\{} length\left({s}_1\right)\\{} length\left({s}_2\right)\end{array}\right\} $$

The values of *Sov* are in the range from 0 to 1, with 0 corresponding to the absence of an overlap and 1 indicating an overlap of a length greater than or equal to one-half of the length of the longer segment. In this study we considered two elements as matching of their *Sov* was equal to 1.

**Figure 1:**
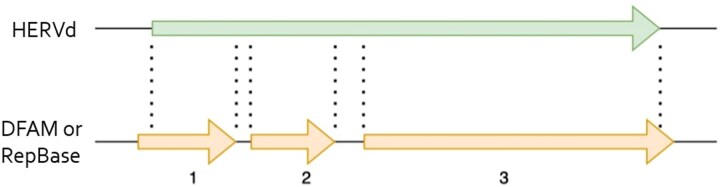
Comparative analysis of repeat annotations. HERVd identifies a large repeat, whereas DFAM and RepBase detect multiple smaller repeats at the same locus.

The three source databases differ in their treatment of elements that become noncontiguous due to insertions and chromosomal rearrangements. While HERVd conducts defragmentation and merges collinear fragments of the same element, DFAM and RepBase rely solely on the initial RepeatMasker hits without further processing. Consequently, a single defragmented element annotated in HERVd may correspond to multiple elements in DFAM and RepBase, as depicted in Fig. 1.

To account for these effects in those cases where Sov < 1.0, we introduced an additional overlap criterion:


$$ len(minov)\ge \textrm{min}\left\{\begin{array}{c}0.95\ast len\left({s}_1\right)\\{}0.95\ast len\left({s}_2\right)\end{array}\right\} $$

which allows for no more than 5% coordinate shift between the two compared elements.

As shown in Fig. 1, HERVd identifies a single large repeat, while DFAM or RepBase detects three smaller repeats within the same locus, likely due to defragmentation. When employing solely the *Sov* criterion to assess concordance between HERVd data and other sources, only the repeat identified by HERVd and the repeat labeled “3” would be flagged as a “match,” while the remaining two would be disregarded due to their size (being less than half the length of the larger HERVd repeat). The second criterion described above ensures that the length of the overlap between two repeats remains ≥95% of the length of the smaller repeat. If this criterion is met, entries with *Sov* below 1 are also classified as matching repeats. Formally, the final criterion for selecting matching records has the form:


$$ \left( Sov=1\right)\vee \left( Sov<1\wedge len(minov)\ge \textrm{min}\left\{\begin{array}{c}0.95\ast len\left({s}_1\right)\\{}0.95\ast len\left({s}_2\right)\end{array}\right\}\right) $$

This approach allows for a broader scope of potential matches to be considered, accommodating instances where the strict *Sov* threshold might have excluded valuable information. A flowchart summarizing the complete analytical workflow for collecting and processing records from data sources is presented in Supplementary Fig. 1.

## Results

### Length distribution and genome coverage of human endogenous retroviral elements annotated in public databases

**Figure 2:**
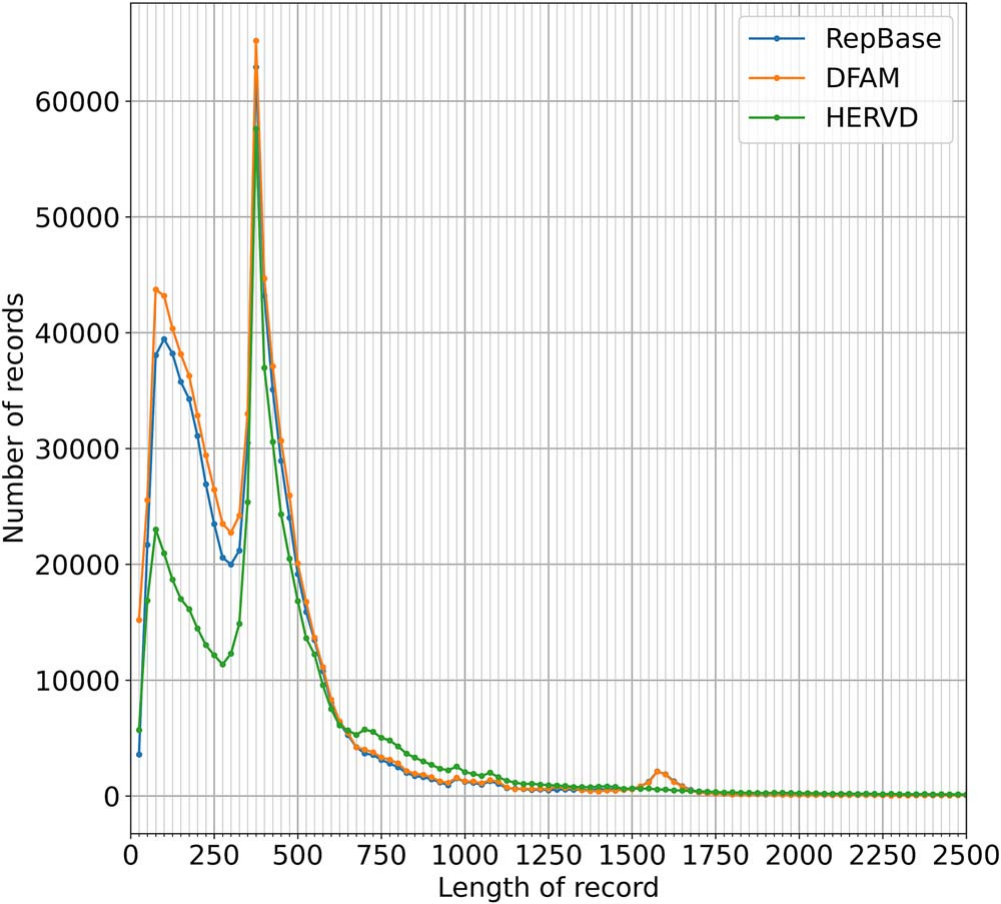
Length distribution of HERV elements in three public databases. Elements longer than 2500 nucleotides (1%–3% of all database entries) are not shown.

We started by investigating the statistical properties of HERV elements annotated in the RepBase, DFAM, and HERVd databases. As seen in Fig. 2, RepBase and DFAM contain about two times more short (fewer than 300 bp) repeats than HERVd, while HERVd contains substantially more longer elements. The main reason for this is semimanual defragmentation of elements in HERVd, which results in several short fragments being combined into one large fragment. Figure 2 also highlights two prominent peaks based on data from all three databases, one in the 200–300 bp region and another in the 400–500 bp region, along with a smaller peak around 1600 bp. The first and second peaks correspond to LTRs associated with ERVs or their remnants. The most numerous LTR families constituting these peaks belong to the MLT1 (J, C, D, K) families. The third peak mostly corresponds to the well-preserved elements from the THE1A (B, D)-int family, which is also extensively present throughout the human genome.

**Figure 3:**
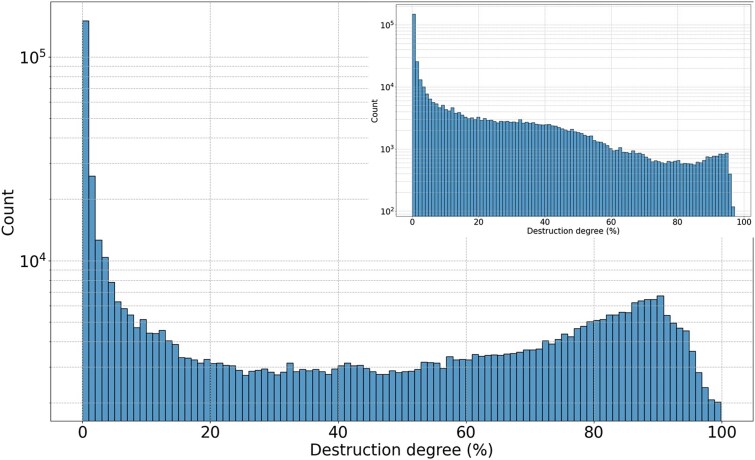
Destruction degree of HERVd entries. The inset shows data for records longer than 250 bp.

Records longer than 2500 bp are excluded from Fig. 2 because they comprise only 1%–3% of all records in each database and would otherwise distort the visual scale. The exact numbers of such entries in DFAM, RepBase, and HERVd are 2453, 4389, and 17 145, respectively. To assess the qualitative characteristics of HERV entries as annotated in the current databases, we referred to the “destruction” parameter available from HERVd, which is calculated relative to the consensus determined for each family of ERV or ERV-associated LTRs (see Materials and methods). As seen in Fig. 3 and Supplementary Fig. 2, the distribution of the destruction degree exhibits two distinct peaks. A significant number of nearly intact entries with a low degree of destruction exist because the consensus is based on these very entries. The second peak, corresponding to significantly destructed elements, mainly consists of small fragments up to 250 bp in length. When examining the same histogram for entries longer than 250 nucleotides, the second peak disappears entirely. Thus, the second peak mostly comprises short, evolutionarily degraded fragments.

**Figure 4:**
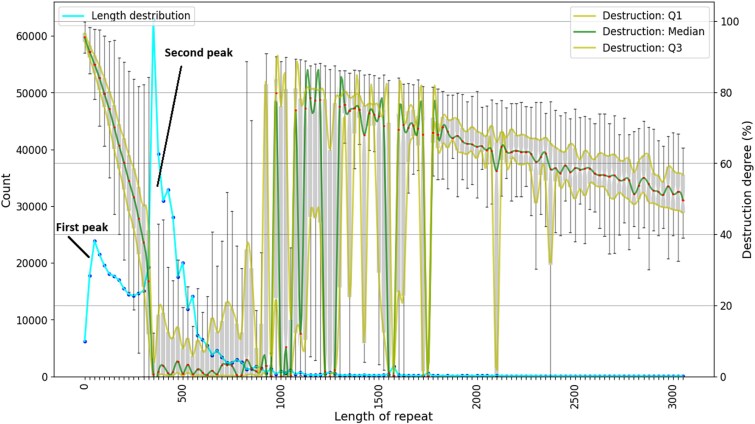
Length and destruction degree (gray box plot) of HERV elements annotated in the HERVd database. Left *y*-axis: The number of records; right *y*-axis: defragmentation degree. Yellow lines on the box plot connect quartiles of destruction degree and green lines connect median values of destruction degree distribution. Elements longer than 3000 nucleotides (1%–2% of all database entries) are not shown.

In an attempt to explain the prominent peaks observed in the length distribution of entries (Fig. 2), we plotted the length of HERV elements together with the degree of their degradation, as shown in Fig. 4. This analysis revealed a sharp decline of destruction values for the entries corresponding to the second peak of the length distribution (length range 300–500 bp specifically for the HERVd database). The destruction curve then sharply rises for entries longer than 1000 bases, because the length of most solo LTRs is below 1000 bp and longer records correspond to less damaged ERVs or intact regions. The curve then exhibits a gradual decline as the length of individual elements approaches the consensus length of the corresponding families. Some exceptions from this tendency are constituted by several sharp dips corresponding to long solo LTRs or internal regions and a bigger dip in the 1500–1600 nucleotide range, which corresponds to the third peak of the length distribution (Fig. 2).

Comparison of length distributions and completeness of HERV elements from individual superfamilies (Supplementary Fig. 3) reveals qualitatively similar trends. Across all superfamilies, three different types of records can be identified. The first region corresponds to degraded solo LTRs, averaging up to 500 bp in length, with the degree of degradation sharply decreasing as the length increases. The second region consists of complete solo LTRs, characterized by a low degree of degradation and a length range between 500 and 1000 bp. The third region encompasses records longer than solo LTRs, representing fragments of larger elements. In this region, the degree of degradation gradually decreases with increasing length, with distinct areas corresponding to intact regions where degradation is almost negligible. These regions will be referenced multiple times in subsequent sections of this chapter.

Intact solo LTRs from the ERV1 family are within the length range 400–1000 bp, while in the ERV2 superfamily solo LTRs are concentrated in two separate regions (around 500 and 1000 bp). The length of the intact solo LTRs belonging to the ERV3 family is within the narrow length range between 500 and 700 bp. The large number of short fragments and the lack of a clear solo LTR zone imply severe disintegration of the Gypsy family elements. Unclassified elements also mostly consist of short elements. Those shorter than 400 bp are heavily damaged, while those 400–1000 bp long have a low damage rate, which corresponds to solo LTR.

The total genome coverage of RepBase (267 268 685 bp) and DFAM (270 488 510 bp) differs by only 2%, while HERVd has a significantly higher coverage of 329 429 968 bp, around 15% higher than the other two data sources. Such lower coverage is likely due to the RepBase data available through the UCSC Genome Browser being outdated. Examining the distribution of the number of records and coverage also reveals family-specific differences (Table 1).

**Table 1:** Number of database records and genome coverage of individual superfamilies; additionally, for HERVd the mean value of destruction degree for each superfamily is given.

Superfamily	DFAM		HERVd			RepBase	
	Number of records	Coverage	Number of records	Coverage	Destruction degree, %	Number of records	Coverage
**ERV1**	214 877	86.1 Mb	119 262	108.2 Mb	34.78	177 905	86.2 Mb
**ERV2**	16 075	10.2 Mb	8427	10.9 Mb	15.79	11 726	9.5 Mb
**ERV3**	511 033	167.0 Mb	408 526	197.3 Mb	32.65	501 207	165.4 Mb
**Gypsy**	20 684	4.3 Mb	18 813	8.8 Mb	73.57	23 966	4.9 Mb
**Unclassified**	19 970	3.7 Mb	10 443	4.1 Mb	68.94	5373	1.2 Mb

**Figure 5:**
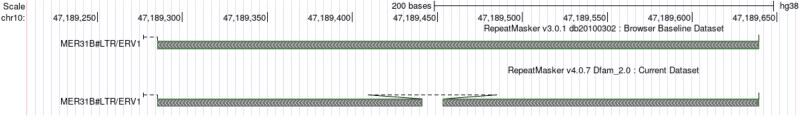
Annotation of the human MERB31B LTR33 repeat in the RepeatMasker and DFAM tracks on the UCSC Genome Browser. According to RepBase, the MERB31B LTR33 repeat comprises a long region of ~350 bp, whereas in DFAM, the same repeat consists of two small regions ~140 and 160 nucleotides in length. Each of these segments is treated by DFAM as a separate record. Such discrepancies cause differences in the number of records between the databases as well as in the total genomic coverage of annotated repeats. The UCSC Genome Browser track labeled “RepeatMasker v3.0.1 db20100302: Browser Baseline Dataset” corresponds to RepBase, while the track titled “RepeatMasker v4.0.7 Dfam_2.0: Current Dataset” corresponds to DFAM.

**Figure 6:**
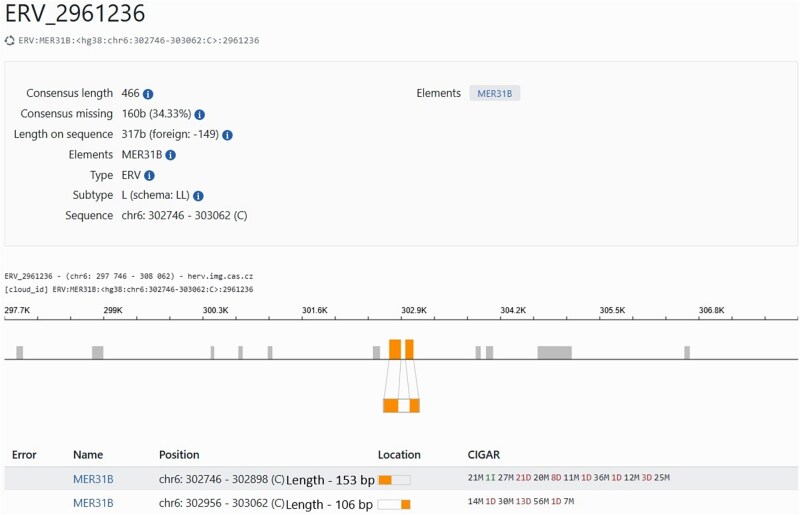
Defragmentation of records in HERVd. The MER31B element is shown as orange boxes while all other repeats in the inspected area of the genome (chromosome 6: 297 746–308 062) are shown as gray boxes. MER31B consists of two fragments, whose coordinates are shown in the table at the bottom of the picture. The total length of the MER31B shown in the box at the top of the picture is greater than the sum of the length of the two fragments as the defragmented element also includes the gap between the two fragments.

For the three major superfamilies—ERV1, ERV2, and ERV3—DFAM, and RepBase, the average length of elements is similar, but there is a much larger number of DFAM elements compared to RepBase. This discrepancy is presumably due to different alignment annotation strategies adopted by these data sources, while the DFAM library is generated by profile HMM searches using nhmmer, RepBase employs Basic Local Alignment Search Tool (BLAST)-like search algorithms (RMBlast or cross_match). Since these search engines have different sensitivities and employ different cut-off thresholds, the same genome region can be aligned differently, which affects the length and the number of fragments detected. Another factor leading to discrepancies is that many complete TEs are broken into multiple DFAM entries representing a portion of the TE, as exemplified in Fig. 5 for MER31B, a member of the ERV1 family. The number of records in the HERVd database is significantly lower due to the defragmentation procedure. It should be emphasized that the defragmentation approach employed in HERVd and the non-fragmented representation of repeats in RepBase represent fundamentally different processes. HERVd applies postannotation defragmentation to merge fragmented repeat instances, whereas RepBase retains consensus repeat sequences without further subdivision into functional components. Additionally, for ERV1 and ERV2, HERVd exhibits higher coverage because defragmentation often results in gap regions between fragments being included into the final coordinates of elements (Fig. 6). This effect is not readily apparent in the figure for ERV3 (Supplementary Fig. 3), presumably due to the large number of very small records (shorter than 250 bp) that cannot be merged. Therefore, the coverage of ERV3 in HERVd is almost identical to that in other databases. As well, we hypothesize that the low coverage of Gypsy records in RepBase may be due to an outdated repeat detection method unable to properly handle the extensive degradation typically associated with this superfamily.

### Record matching

After evaluating the overall composition of each database, we proceeded to compare their individual records. The primary tool for this task was the Python package PyRanges, which enables efficient processing and comparison of tables containing large numbers of intervals. In the first step, we compared records based on the *Sov*, which represents the degree of overlap between two intervals, ranging from 0 to 1, with a maximum value indicating an overlap equal to or greater than half the length of the longer interval (see the Materials and methods section).

Table 2 illustrates that 15%–49% of each data source is unique, with no overlap at Sov = 1. DFAM and RepBase show the highest degree of overlap (~85%) with each other, while their overlap with HERVd is ~50%. Meanwhile, around 70% of HERVd records are shared with at least one other database. In contrast, around half of the entries in DFAM and RepBase are found across all three databases, whereas 68% of HERVd elements appear in all of them. Overall, ~20% of DFAM and HERVd dataset remains unique to its respective source, while for RepBase this value is 8%.

**Table 2:** The number of matching records and their total length, while comparing data sources with Sov set to 1.

Target database/source database	DFAM	HERVd	RepBase
DFAM	782 639 (100%), 271 Mb (100%)	405 703 (71.7%), 156 Mb (46.9%)	630 224 (80.5%), 230 Mb (85.0%)
HERVd	405 003 (51.7%), 154 Mb (56.7%)	565 471 (100%), 329 Mb (100%)	414 633 (57.6%), 162 Mb (60.8%)
RepBase	630 232 (87.5%), 232 Mb (86.8%)	415 414 (73.5%), 166 Mb (50.0%)	720 177 (100%), 267 Mb (100%)
Present in each database	386 084 (49.3%), 148 Mb (54.7%)	386 626 (68.4%), 149 Mb (45.1%)	386 818 (53.7%), 150 Mb (56.1%)
Unique elements	133 496 (17.1%), 35 Mb (13.0%)	130 980 (23.1%), 159 Mb (48.2%)	62 130 (8.6%), 23 Mb (8.5%)

Regarding the total coverage (the combined length of all records), we observe a 50% difference between HERVd and either DFAM or RepBase, and a 15% difference between DFAM and RepBase. Despite these variations, roughly half of the total coverage is represented in all three databases, and 15%–50% of each database’s coverage is absent in pairwise comparisons. Notably, even with substantial differences in the total number of records, the overall proportion of matching records remains relatively consistent across all comparison groups.

The low consistency between the databases, especially when comparing HERVd to others using only ‘Sov,’ arises from the postprocessing steps applied after repeat identification. HERVd performs defragmentation by merging multiple fragments of a single damaged HERV at a given chromosomal location. As a result, if the length of even a single fragment is less than half the size of the defragmented record, the *Sov* value for that overlap is guaranteed to be <1.

Since it is more important to establish the correspondence between biologically relevant HERV elements than to formally match database records, we introduced an additional parameter that marks two overlapping repeats as “matched” if one of them overlaps by more than 95% with the other one, for Sov < 1 (see Materials and methods section). Using these more stringent matching criteria leads to a substantially improved consistency between the three data sources (Table 3), with 88%–93% of corresponding records and the percentage of unique elements varying between 1.2% and 4.3%. Similarly, the differences in genome coverage decreased to 2%–7%. Notably, the proportions of matching records across all pairwise comparisons now correspond to the ratios between the total number of records in each database. These findings indicate that the primary reason for the observed discrepancies lies in the distinct methodologies used to define ERVs. HERVd tends to join multiple HERV fragments, while DFAM and RepBase define each fragment as a separate record. We investigated in more detail two particularly interesting groups of records: those shared across all databases and those exclusive to each individual database. Using the improved matching strategy, at least 88% of records are shared between any pair of databases (Table 3).

**Table 3:** The number of matching records and their total length while comparing data sources with Sov = 1 or with Sov < 1 and an additional parameter which allows for no more than 5% coordinate shift between the two compared elements.

Target database/source database	DFAM	HERVd	RepBase
DFAM	782 639 (100%), 271 Mb (100%)	540 493 (95.6%), 323 Mb (98.0%)	688 004 (95.5%), 262 Mb (98.2%)
HERVd	714 183 (91.3%), 259 Mb (95.4%)	565 471 (100%), 329 Mb (100%)	681 315 (94.6%), 258 Mb (96.6%)
RepBase	725 552 (92.7%), 264 Mb (97.2%)	541 962 (95.8%), 322 Mb (97.7%)	720 177 (100%), 267 Mb (100%)
Presented in each database	691 079 (88.3%), 254 Mb (93.7%)	526 084 (93.0%), 318 Mb (96.5%)	658 078 (91.4%), 255 Mb (95.2%)
Unique elements	33 983 (4.3%), 3 Mb (1.2%)	9100 (1.6%), 3 Mb (0.9%)	8936 (1.2%), 1 Mb (0.4%)

**Figure 7:**
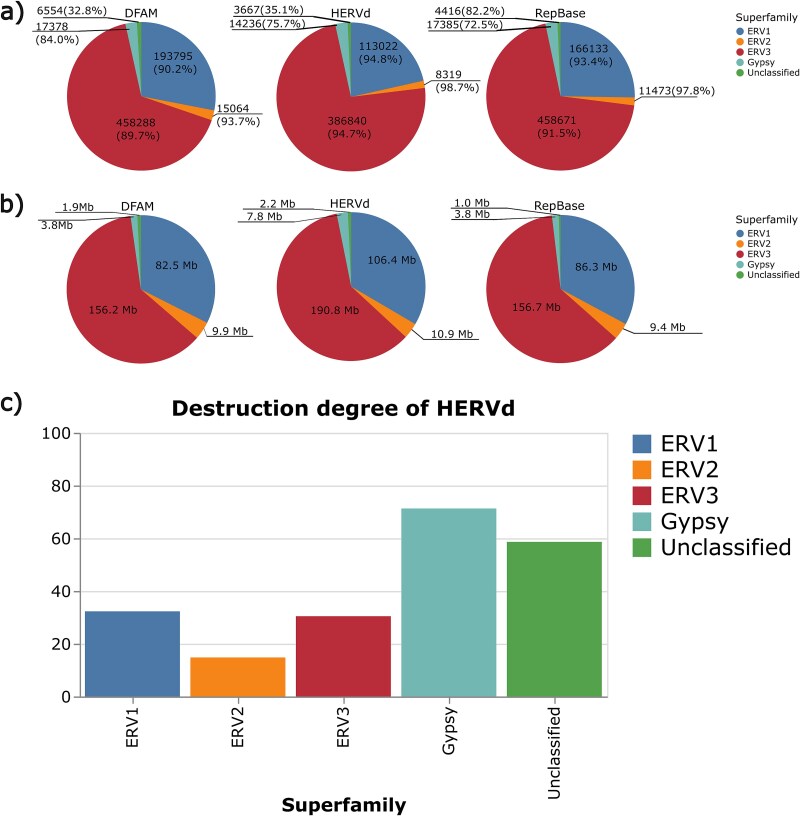
Records shared between all data sources and their genomic coverage. (a) Number of records and their percentage of the total in each database, shared across all databases and categorized by superfamilies. For example, 83.1%–90.2% of all ERV1 records annotated in the DFAM database are shared with both HERVd and RepBase. (b) Total length of the shared records. For example, 82.5 and 79.4 Mb of ERV1 records annotated in the DFAM are shared with both HERVd and RepBase. Because the overall differences in the number of records and coverage among the data sources are minimal, the corresponding chart sizes appear almost identical. (c) Destruction degree for HERVd records shared between all data sources, categorized by superfamilies.

Given the strong concordance among data sources, one might anticipate that the distribution of records across individual superfamilies would reflect their overall prevalence. For example, if 80% of DFAM entries appear in the other two databases, one would expect 80% of DFAM’s ERV1 elements to likewise be present elsewhere. However, as illustrated in Fig. 7, the correspondence between data sources, both in terms of element count and genomic coverage, varies considerably across different subfamilies. The EVR1, ERV2, and ERV3 families are rather evenly represented in all three sources. By contrast, ~84% of DFAM’s Gypsy records are present in the other databases, whereas only 72.5% of RepBase’s and 75.7% of HERVd’s Gypsy elements are represented (Fig. 7a), probably because of their high degree of destruction. On the other hand, 82.2% of unclassified records from RepBase are also found in the other two databases. In contrast, only 32.8% of DFAM’s unclassified elements and 35.1% of HERVd’s are presented in each database, likely because some of these records were assigned to different superfamilies by DFAM and HERVd and also because DFAM and HERVd are able to classify ERVs more accurately due to improved methodology (Fig. 7a).

When examining genomic coverage, the distribution among the three databases follows a somewhat narrower range than the overall pattern (Fig. 7b). Overall, HERVd exhibits the highest genomic coverage, whereas RepBase, closely followed by DFAM, shows slightly lower total coverage. At the superfamily level, all records, except for unclassified ones, follow a similar trend. For example, ERV2 coverage is nearly identical in DFAM (9.9 Mb) and RepBase (9.4 Mb), whereas in HERVd it is slightly higher (10.9 Mb). ERV1, ERV3, and Gypsy display comparable patterns, with coverage in HERVd being moderately higher than in RepBase and DFAM. In contrast, unclassified repeats show the opposite trend, with HERVd and DFAM covering the largest portion relative to the RepBase database.

One would expect the proportion of matched records across databases, relative to the total number of records for each database, to remain stable within superfamilies. Indeed, ERV1 and ERV3 adhere to this assumption: the proportion of records across all five families from each database that are found in all sources is 88.3%, 93.0%, and 91.4% for DFAM, HERVd, and RepBase, respectively (Table 3). The proportions of total superfamily entries for ERV1 and ERV3 closely align with the overall trends, amounting to 90.2% and 89.7% for DFAM, 94.8% and 94.7% for HERVd, and 93.4% and 91.5% for RepBase. ERV3 and ERV1 cover the largest proportion of records, so the majority of records fulfill the assumption described above. However, this pattern does not extend to other superfamilies. The proportion of ERV2 entries represented in all databases is higher than the overall distribution: 93.7% for DFAM, 98.7% for HERVd, and 97.8% for RepBase. These discrepancies are due to the previously discussed differences in repeat detection methods, as well as the influence of the degree of degradation on the identification of repeat boundaries. RepBase differs from other databases in that a significant portion of unclassified RepBase entries is represented in other databases with approximately the same coverage as DFAM and HERVd. At the same time, RepBase demonstrates comparable coverage and number of entries in other categories, which is likely the result of an older detection approach. This indicates that RepBase did indeed identify and classify these repeats, but there are clearly repeats that can only be identified using modern methods. Meanwhile, the differences between DFAM and HERVd largely reflect the defragmentation process described earlier.

**Figure 8:**
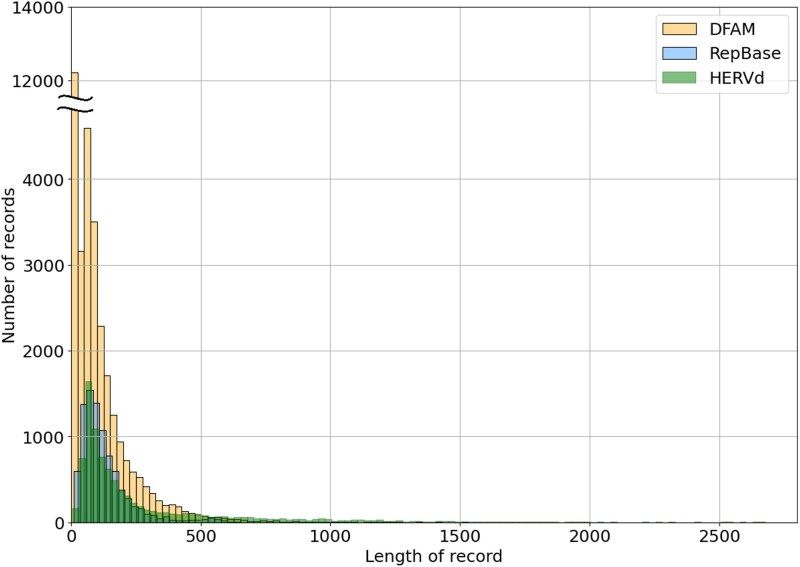
Length distribution of unique records for each data source.

**Figure 9:**
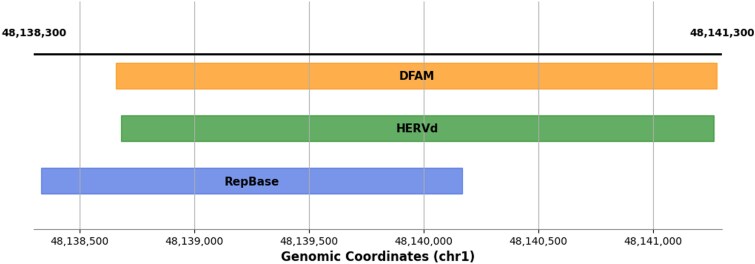
The LOR1-int repeat, which is labeled as unique in RepBase. The figure shows the location of this repeat according to RepBase (chr1:48 138 333–48 140 169, length 1836 bp) relative to its counterparts in HERVd (chr1:48 138 680–48 141 264, length 2584 bp) and DFAM (chr1:48 138 659–48 141 279, length 2620 bp). The Sov value for the overlap between the RepBase repeat and its counterparts in HERVd and DFAM is around 0.8—below the threshold of 1—and it is shifted by ~300 bp downstream, exceeding 5% of the total repeat length. Consequently, this overlap does not satisfy the criterion for selecting matching records described in the Materials and methods. This example illustrates how differences in repeat annotation can arise from improved detection algorithms.

The percentage of unique records varies significantly across data sources, with 1.6% in HERVd, 1.2% in RepBase, and 4.3% in DFAM (Table 3). As illustrated in Fig. 8, unique records in HERVd are very short, rarely exceeding 200 bp. Unique records in DFAM are also relatively short, with the majority ranging from 0 to 500 bp. In RepBase, the distribution primarily consists of short entries, though it also contains a small subset of longer records. The predominance of shorter entries among the unique records in each database arises from the inherent difficulty of detecting them. Meanwhile, the presence of longer entries in RepBase can be attributed to its relatively outdated repeat-identification method, as illustrated in Fig. 9.

**Figure 10:**
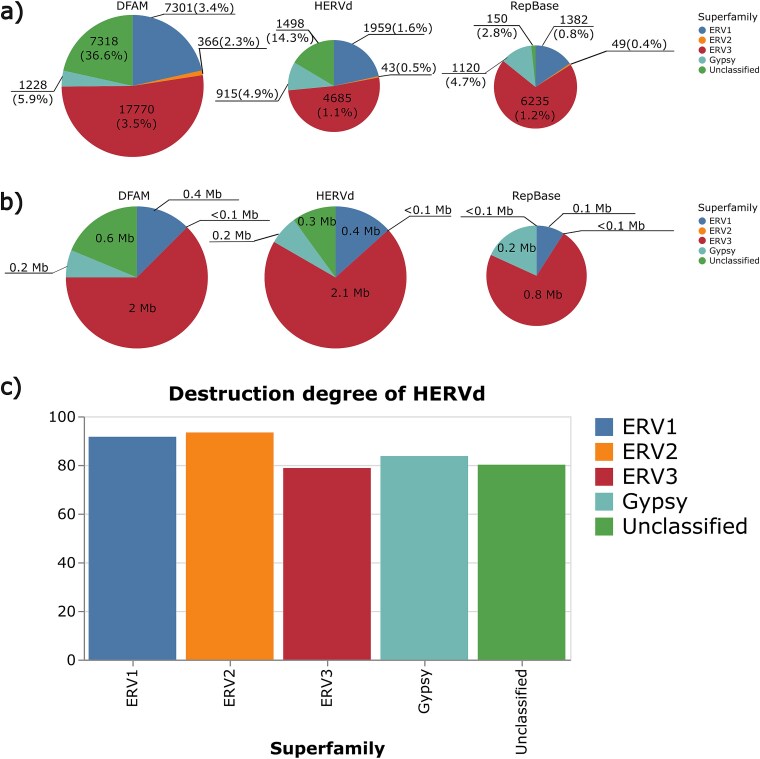
Records unique for each data source and their genomic coverage. (a) Number of unique records and their percentage of the total in each database, categorized by superfamilies. (b) Total length of unique records. The relative chart sizes reflect the overall proportions of the number of records or coverage across the data sources. Given the substantial differences (DFAM: 33 983–70 000 records and 3 18 Mb; HERVd: 9100–14 000 records and 35 Mb; RepBase: 8936–55 000 records and 1 19 Mb), the charts also vary considerably in size. (c) Destruction degree of unique HERVd records, categorized by superfamilies.

The total number of unique records differs strongly between the three databases, amounting to 9100 in HERVd, 8936 in RepBase, and 33 983 in DFAM. This imbalance is not evenly distributed across superfamilies (Fig. 10). For ERV1, the number of unique records in RepBase (1382 records) and HERVd (1959 records) is markedly lower than in DFAM (7301 records) (Fig. 10a). By contrast, the total coverage of ERV1 in DFAM (0.4 Mb) and HERVd (0.4 Mb) is higher than in RepBase (0.1 Mb) (Fig. 10b). A similar pattern is observed for ERV3: DFAM (17 770 records, 2.0 Mb) also shows a greater number of unique entries and higher coverage than RepBase (6235 records, 0.8 Mb) and similar coverage compared to HERVd (4685 records, 2.1 Mb). However, for ERV2, the number of entries is so small that the total coverage is <0.1 Mb. HERVd (43 entries) and RepBase (49 entries) have similar metrics, unlike DFAM (366 entries). Unique Gypsy entries are equally represented in all sources: DFAM (1228 records, 0.2 MB) has only slightly more such elements than RepBase (1120 records; 0.2 MB) and HERVd (915 records, 0.2 MB). DFAM contains the highest number of unique Unclassified elements (7318 records, 0.6 Mb), five times more than in HERVd (1498 records, 0.3 Mb) and 50 times more than in RepBase (150 records, <0.1 MB). According to HERVd, these entries are highly degraded (Fig. 10a), making it difficult to correctly identify their boundaries. Therefore, the coordinates of the records in question do not overlap between databases. These results are consistent with Fig. 8, where DFAM contains predominantly very short fragments, but the overall coverage is almost identical to HERVd, while RepBase contains fewer entries with a total length that is three times shorter.

## Discussion

During this analysis, three of the most frequently used data sources for annotating HERV: RepBase, DFAM, and HERVd, were examined. The comparison was carried out based on the coordinates of ERV and ERV-associated LTR elements, considering the postprocessing criteria specific to each database. RepBase is a well-established and widely used project that pioneered systematic annotation of eukaryotic transposable elements a quarter of a century ago [18], but the open-access version of this resource has not been updated for more than a decade. DFAM, by contrast, prioritizes the development and refinement of methods for detecting repetitive elements using hidden Markov models rather than providing comprehensive annotation for a specific species. Consequently, while DFAM offers current tools and libraries, the depth of information for individual genomes may be limited. In contrast, HERVd, another well-established and long-running initiative [7], is dedicated to the detailed, semimanual annotation of human HERVs exclusively, ensuring more exhaustive coverage of individual records.

Notable differences among these three sources emerged when evaluating both the number and total length of overlapping elements. These discrepancies can be attributed not only to variations in filtering algorithms and different versions of the databases, but also to divergent postprocessing strategies and merging criteria for genomic coordinates. In the publicly available version of RepBase, the primary issue is the age of the last update; DFAM employs highly effective methods for repeat detection but does not always offer in-depth annotation for individual genomes; HERVd, on the other hand, performs additional “defragmentation” of records, reducing the overall number of identified elements without diminishing their biological relevance, and provides more detailed information on each specific repeat.

Data retrieval from these three distinct sources proved challenging due to the absence of a unified data format, diverse versioning practices, and varying access requirements, making direct comparisons more difficult. Nonetheless, the results clearly indicate that each resource has its own domain of applicability: RepBase (especially the current public version) may be useful for certain projects that do not require the very latest annotation, DFAM provides state-of-the-art tools for constructing and testing models for repeat detection, and HERVd offers comprehensive human-genome annotation, which can be indispensable for research focusing specifically on humans. Conducting self-annotation using DFAM libraries or other open-source tools can be an effective approach for researchers seeking maximum flexibility and up-to-date data, although this requires additional computational resources and technical expertise.

Thus, there is currently no single “best” resource for HERV annotation. Each database examined here possesses notable advantages and limitations that should be considered when selecting the most appropriate tool for a given research objective. While our primary goal was to benchmark and validate existing HERV resources, we did not release a new unified annotation, as doing so would require sustained curation and versioning infrastructure beyond the scope of this study. For unified, locus-resolved human ERV annotation, we recommend HERVd, which is tailored to ERV annotation at the genomic locus level. If you need to identify repetitions in your own data, DFAM with its HMMs is perfect for this. All were using the GRCh38/hg38 reference genome. With the advent of the telomere-to-telomere assembly (T2T-CHM13 [19]) the community now has access to a more complete human genome sequence, yet dedicated libraries and curated annotations for mobile elements, including HERVs, have not yet been generated for this build. By highlighting this diversity, the present study contributes to a deeper understanding of the current state of HERV annotation and may serve as a starting point for future work aimed at developing unified approaches and standards in the field.

**Key Points**

- Human endogenous retroviruses (HERVs) are increasingly recognized for their significant roles in human biology and disease, yet annotations remain inconsistent across major databases (DFAM, HERVd, RepBase).
- Annotation discrepancies primarily result from methodological differences: DFAM uses automated detection with hidden Markov models, HERVd employs detailed semimanual defragmentation, and RepBase’s freely available annotations date from 2013.
- Despite differences, refined matching criteria allow reconciliation of up to 93% of annotated HERV elements, although each database maintains unique annotations.
- Researchers should strategically select annotation resources based on project needs: DFAM for computational flexibility, HERVd for detailed human-specific annotations, and RepBase for projects not requiring the latest annotation updates.
- This comparative analysis highlights the critical need for harmonized HERV annotation standards, particularly in the context of emerging complete genome assemblies such as T2T-CHM13.

## Supplementary Material

Supplementary_figure_12_10_25_bbag062

Supplementary_file_1_12_10_25_bbag062

## Data Availability

Raw datasets from the three sources (HERVd, DFAM, and RepBase), along with the family–superfamily mapping table used in this study, have been deposited on Zenodo [https://doi.org/10.5281/zenodo.15053079]. These files include IDs, genomic coordinates, and the initial classifications of HERV elements into families. Note that the original databases do not provide associations between the internal portions of HERVs, their LTRs, and superfamilies; these associations were established as described in our previous work [15].
