# Supplementary material for: Current status of human endogenous retrovirus annotation
Source: Brief Bioinform. 2026 Feb 16;27(1):bbag062. doi: 10.1093/bib/bbag062 (PMC12907019; doi:10.1093/bib/bbag062)
Supplement: Supplementary_figure_12_10_25_bbag062 [file supplementary_figure_12_10_25_bbag062.docx]

Supplementary information


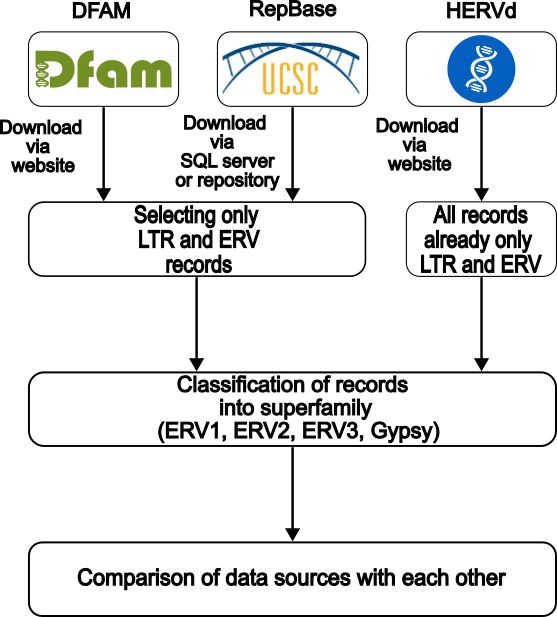


Supplementary figure 1. A flowchart summarizing the complete analytical workflow for collecting and processing records from data sources.


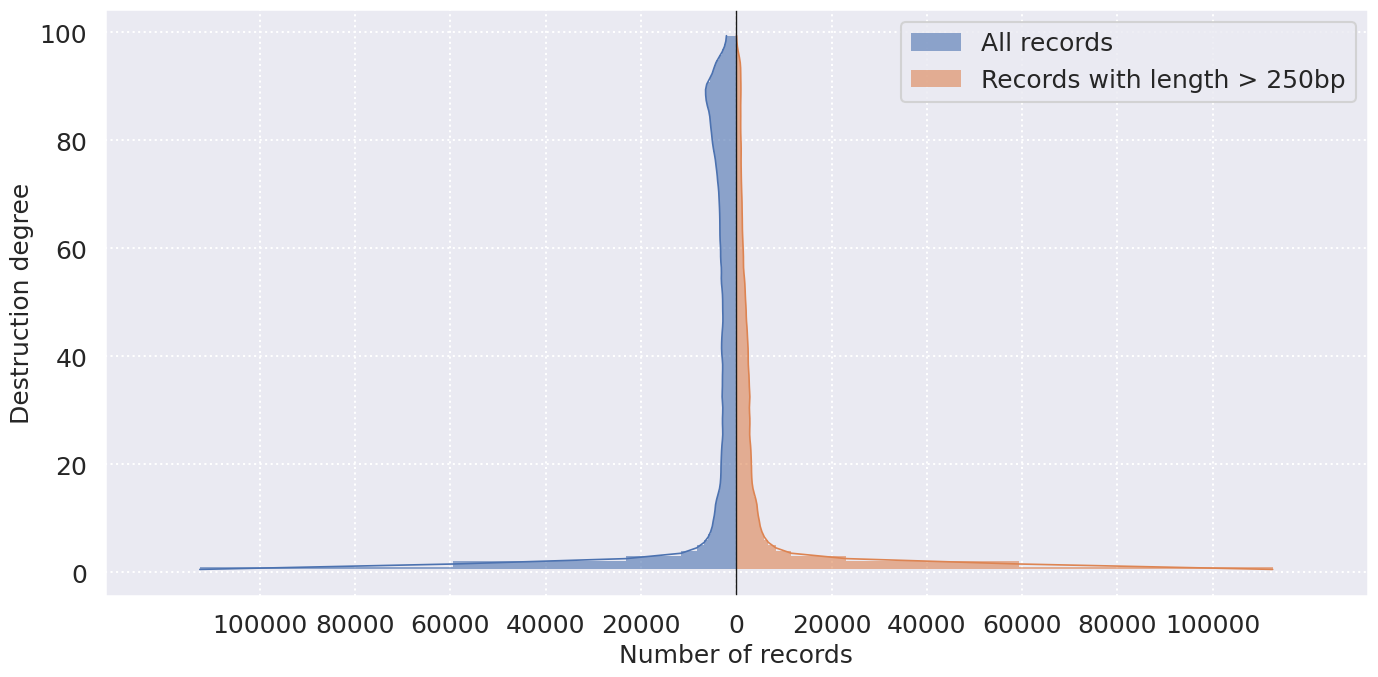


Supplementary figure 2. Violin plot of HERV element lengths from HERVd shown for all entries – the blue one (n = 565,471) and for entries >250 bp – the orange part (n = 407,050).


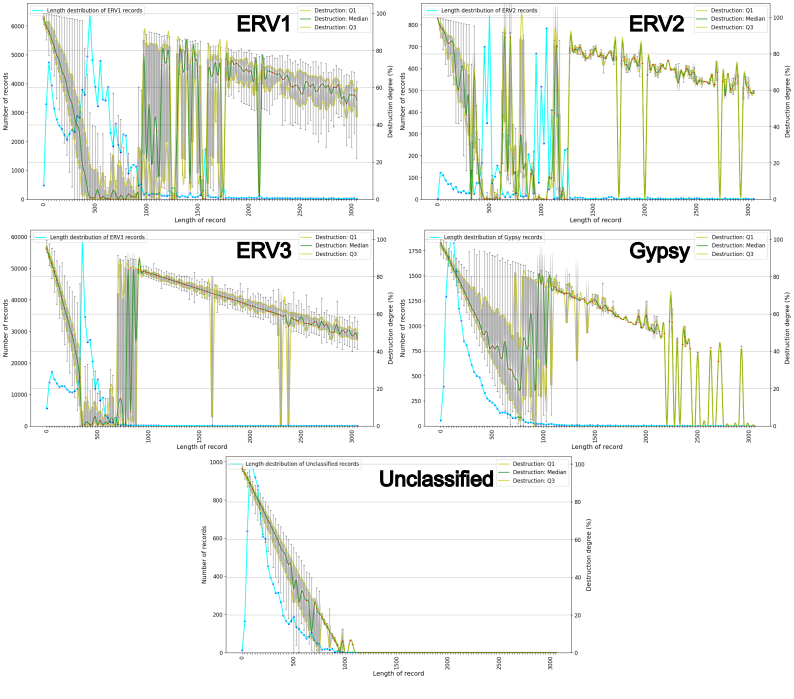


Supplementary figure 3. Length (magenta line) and destruction degree (grey box plot) for individual HERV families based on HERVd annotation. Left Y axis: the number of records; right Y axis: defragmentation degree. Yellow lines on the box plot connect quartiles of destruction degree and green lines connect median values of destruction degree distribution. Elements longer than 3000 nucleotides (1-2% of all database entries) are not shown.
