# Supplementary material for: Current status of human endogenous retrovirus annotation
Source: Brief Bioinform. 2026 Feb 16;27(1):bbag062. doi: 10.1093/bib/bbag062 (PMC12907019; doi:10.1093/bib/bbag062)
Supplement: Supplementary_file_1_12_10_25_bbag062 [file supplementary_file_1_12_10_25_bbag062.docx]

Data collection manual

RepBase (via UCSC)

Important note: The most recent public version is currently available through the UCSC Genome Browser, while ongoing updates are accessible via subscription.

There are several ways to collect data from UCSC’s tracks, first – via SQL server, we used this one, but we recommend the easier way – via their repository directly.

For SQL here is the UCSC manual: <https://genome.ucsc.edu/goldenPath/help/mysql.html> .

The same file you can obtain from this repository: <https://hgdownload.soe.ucsc.edu/goldenPath/hg38/bigZips/> .

In our comparison we used this annotation which is the lasted open access version of annotation made with RepBase data source:
“hg38.fa.out.gz - RepeatMasker .out file. RepeatMasker was run with the

-s (sensitive) setting.

June 20 2013 (open-4-0-3) version of RepeatMasker

RepBase library: RELEASE 20130422”

You can obtain it with this command:

**wget** [**https://hgdownload.soe.ucsc.edu/goldenPath/hg38/bigZips/hg38.fa.out.gz**](https://hgdownload.soe.ucsc.edu/goldenPath/hg38/bigZips/hg38.fa.out.gz)

HERVd

All annotation available on their website here: <https://herv.img.cas.cz/downloads> .
In our comparison we used this annotation: Package for ERV (bed.gz) - <https://herv.img.cas.cz/f/package-entities-erv.bed.gz>

DFAM

Latest available DFAM annotation you can find here:
<https://dfam.org/releases/Dfam_3.8/annotations/hg38/hg38.hits.gz>
